# Genotoxic exposures to volatile organic compounds in golden retrievers with and without multicentric lymphoma

**DOI:** 10.3389/fvets.2026.1783854

**Published:** 2026-04-13

**Authors:** Ashleigh N. Tindle, Lauren M. Krueger, Brenna Swafford, Julia Labadie, Lauren A. Trepanier

**Affiliations:** 1 Department of Medical Sciences, University of Wisconsin-Madison, Madison, WI, United States; 2 Scientific Programs Department, Morris Animal Foundation, Denver, CO, United States

**Keywords:** cancer risk, canine, genotoxicity, lymphoma, volatile organic compounds (VOCs)

## Abstract

Canine multicentric lymphoma is a common cancer in dogs with poor long-term outcomes. In people, non-Hodgkin lymphoma is associated with volatile organic compounds (VOCs), but this risk is not clear for lymphoma in dogs. The objective of this study was to investigate whether dogs with lymphoma were more likely to be exposed to DNA-damaging concentrations of VOCs compared to unaffected controls. We measured stable urinary metabolites of the VOCs benzene, xylene, and 1,3-butadiene in golden retrievers with multicentric lymphoma and matched unaffected controls at 2 time points: the time of diagnosis and 1 year prior. We then determined genotoxic effects for xylene and bioactivated benzene in canine peripheral blood mononuclear cells (PBMCs) *in vitro* and compared them to *in vivo* blood concentrations estimated using reverse dosimetry. All dogs had detectible urinary concentrations of the benzene metabolites PHMA and MUCA, the xylene metabolite 34MHA, and 1,3-butadiene metabolites MHB3 and DHBM, with no differences between cases and controls. Six of 30 cases and none of 30 controls were positive for urinary cotinine, a marker of secondhand smoke (*p* = 0.028), but urinary VOC concentrations did not differ between cotinine-positive and -negative dogs. Bioactivated benzene was genotoxic to canine PBMCs at ≥ 10 uM and xylene was genotoxic at ≥ 0.5 uM. Estimated blood benzene and xylene concentrations reached genotoxic exposures in most dogs. These results support a recommendation to reduce VOC exposures in pet dogs, for example by using activated carbon indoor air filtration units rated for VOC removal.

## Introduction

Canine multicentric lymphoma is a common cancer in pet dogs, affecting many thousands of dogs per year (1). Canine lymphoma (CL) can be treated with multimodal chemotherapy, but relapses occur in more than 90% of cases, and dogs are typically euthanized for recurrent disease within a year of diagnosis (2). While certain breeds such as golden retrievers have a higher risk for lymphoma, environmental chemicals also appear to play a role (3, 4). A better understanding of environmental risk factors for lymphoma in dogs might help reduce the incidence of this devastating cancer.

Multicentric lymphoma in dogs shares clinical features with human non-Hodgkin lymphoma (NHL) (5). Canine lymphoma and NHL can even share common geographic distributions (6), suggesting possible shared environmental risk factors for lymphoma in dogs and people.

Human lymphoid malignancies such as NHL are linked to occupational exposures to volatile organic compounds (VOCs) such as benzene, xylene, and 1,3-butadiene (7, 8), as well as to community-based exposures to these chemicals (9). Furthermore, benzene and 1,3-butadiene cause lymphoma in mice (10, 11), and benzene, xylene, and 1,3-butadiene, or their metabolites, are genotoxic to human lymphoid cells (12–14).

In dogs, lymphoma has been associated epidemiologically with household use of VOC-containing paints and solvents (3) and proximity to industrialized or polluted sites (3, 4), which are also common sources of VOCs. However, dogs with lymphoma have not been directly bio monitored for VOC exposures, and these chemicals have not been assessed for genotoxicity toward canine lymphoid cells. As such, it was not known whether DNA-damaging exposures to specific VOCs were associated with multicentric lymphoma in dogs.

Our overall hypothesis was that lymphoma in dogs is associated with genotoxic exposures to specific volatile organic compounds. To address this hypothesis, we conducted a case–control study of multicentric lymphoma in pet golden retrievers to: (1) compare stable urinary metabolites of benzene, xylene, and 1,3-butadiene; (2) assess the genotoxicity of VOCs toward canine lymphoid cells *in vitro* using the alkaline CometChip assay; and (3) use reverse dosimetry to estimate blood VOC concentrations from urinary metabolites and determine whether dogs with lymphoma have greater estimated genotoxic VOC exposures compared to controls. The overall goal was to identify potentially modifiable chemical exposures associated with lymphoma in dogs.

## Methods

### Cases and controls

We used a nested case–control study design that leveraged banked urine samples from an existing longitudinal cohort, the Golden Retriever Lifetime Study (GRLS) (15). Dogs included in the GRLS cohort were purebred golden retrievers that were enrolled from 6 months to 2 years of age between 2012 and 2015, and followed with yearly vet appointments, owner questionnaires, and annually banked samples of blood and urine. Our case population was 30 randomly selected golden retrievers that had been diagnosed with multicentric lymphoma from 2015 to 2022. Dogs with multicentric lymphoma were diagnosed by cytology or histopathology reviewed by a board-certified veterinary pathologist; immunophenotyping was not required but was included when available. Dogs with primary gastrointestinal, cutaneous, or neurologic manifestations of lymphoma were excluded.

Our control population was unaffected golden retrievers from the same cohort that were matched to cases by age, sex/neuter status, and a 1–2-month sampling window (Table 1) (16). Control dogs underwent annual complete physical exams and screening bloodwork through the GRLS longitudinal monitoring protocol. No control dogs developed lymphoma in the 5 years after sampling for this study.

**Table 1:** Demographic characteristics of a case–control study of golden retriever dogs with multicentric lymphoma assessed for volatile organic compound (VOC) exposures.

Demographic	Multicentric lymphoma cases	Unaffected controls
Number of dogs	30	30
Age at diagnosis or matched enrollment (median and range)	7.1 years (3.5–10.6)	7.1 years (3.2–10.9)
Sex and neuter status	FS 8 FI 4 MN 12 MI 6	FS 8 FI 4 MN 12 MI 6
Immunophenotype of lymphoma	B cell, overall, 14 T cell, overall, 11 Not characterized 5	Not applicable

FS, female spayed; FI, female intact; MN: male neutered; MI, male intact.

For our study, we had access to urine samples from the time of lymphoma diagnosis (T0) and 1 year prior to diagnosis (T-1y) for cases, and from comparable dates for matched controls. Most cases and controls (93% each) remained at the same home address over the 2-year sampling period.

### Urinary VOC metabolites

Major urinary metabolites of benzene, xylene, and 1,3-butadiene were measured at T0 and T-1y in 1 mL of voided urine from each dog. Samples were frozen at -80 °C after collection and shipped on dry ice to the Centers for Disease Control Volatile Organic Compounds (CDC) VOC Laboratory. The metabolites PHMA (N-acetyl-S-(phenyl)-L-cysteine) and MUCA (muconic acid) were measured for benzene; 3,4-MH (3-methylhippuric acid + 4-methylhippuric acid) was measured for xylene, and MHB3 (N-acetyl-S-(4-hydroxy-2-buten-1-yl)-L-cysteine) and DHBM (N-acetyl-S-(3,4-dihydroxybutyl)-L-cysteine) were measured for 1,3-butadiene. All VOC metabolites were quantified by ultra-performance liquid chromatography with electrospray tandem mass spectrometry (method DLS 2103a) (17) by the CDC VOC Laboratory, on a fee-for-service basis. Individual assay results were reviewed for quality control by a quality assurance officer at the CDC before release. All analytes were normalized to urine creatinine, measured by ELISA (BioAssay 89 Systems QuantiChromTM Creatinine Assay Kit DICT-500), to control for individual differences in urine concentration.

### Urinary cotinine

Urinary cotinine (a metabolite of nicotine) was measured in all cases and controls at both time points to assess exposure to secondhand smoke as a possible source of VOCs. Cotinine was measured using a solid-phase competitive ELISA method (Abnova Cotinine ELISA, KA0930 v08) with a limit of detection of 5 ng/mL. Because benzene, xylene, and 1,3-butadiene have all been detected in tobacco smoke (18, 19), we also compared aggregate urinary molar VOC metabolite concentrations between dogs with positive and negative urinary cotinine at either time point.

### Household environmental questionnaire responses

Owners of enrolled dogs in the GRLS were asked to complete questionnaires about household exposures at annual sample collection times. We compared aggregate molar urinary VOC metabolites based on responses to two questions relevant to VOC exposures: 1) the type of stove used for cooking (natural gas or propane versus electric), and 2) whether a wood fireplace or wood-burning stove was used in the home. Aggregate molar VOC metabolite concentrations were calculated by converting urinary concentrations in ng/mL to nM using the molecular weights of PHMA (239.3 g/mol), MUCA, (142.1 g/mole), 34MHA (193.2 g/mole), MHB3 (233.3 g/mole) and DHBM (251.3 g/mole) and adding them together for each dog at either time point.

### Estimated blood VOC exposures

We used reverse dosimetry (20) to estimate blood VOC exposures from observed urinary concentrations measured in cases and controls. Reverse dosimetry was based on available data for percent urinary excretion for each metabolite and volume of distribution for the parent chemicals (Table 2), using the following equations:

1) Internal “dose” of metabolite (umole) = Unadjusted urine concentration (converted to umole /L) × estimated daily urine volume (0.030 L/kg* kg body weight) (25). Individual kg body weights were used for each dog.
2) Estimated amount of original parent VOC in body = Internal “dose” (umole) of metabolite / urinary excretion factor for each metabolite (Table 2).
3) Estimated blood concentration of parent VOC (umole/L) = Estimated concentration of parent compound in body / Vd (volume of distribution in L/kg *kg body weight), Table 2).

**Table 2:** Reverse dosimetry assumptions used to estimate whole blood VOC (volatile organic compound) concentrations from measured urinary volatile organic compound (VOC) metabolites in pet dogs.

Parent VOC	Estimated volume of distribution	Urinary metabolite	Urinary excretion factor
Benzene	0.011 L/kg (21) (data from rats)	PHMA *N*-acetyl-S-(phenyl)-L-cysteine	0.11% (22) (data from humans)
		MUCA Muconic acid	3.9% (22) (data from humans)
Xylene	0.03 L/kg (23) (data from humans)	3MHA (*m*-) 3-Methylhippuric acid	*m-*xylene: 72% (24) (data from humans)

The urinary excretion factor is the percentage of parent VOC that appears in urine as the designated metabolite.

### *In vitro* DNA damage

To assess concentration-dependent DNA damage for VOCs in dogs, we used the *in vitro* CometChip assay, a high-throughput version of the traditional alkaline comet assay that reduces intra-assay variability. This assay measures both single and double-stranded DNA breaks. All CometChip experiments were performed using a standardized protocol (26); the assay developer, Dr. Bevin Engelward, generously provided our laboratory with our own CometChip “stamp,” which generates microwells in 96 well plates that aid in even cell distribution.

We chose normal canine peripheral blood mononuclear cells (PBMCs) as target cells for genotoxicity assessments. For PBMC preparation, fresh whole blood samples in EDTA were collected from a pool of 6 healthy dogs of various breeds belonging to faculty/staff at the University of Wisconsin-Madison School of Veterinary Medicine. These included 3 female spayed and 3 male neutered dog ranging in age from 1 to 6 years. Blood was collected with informed consent under institutional IACUC approval. Cells (PBMCs) were prepared by standard density centrifugation at 400 x *g* for 30 min in Histopaque 1,077 (Sigma Aldrich Cat No. 10771). Cell pellets were washed and resuspended in 1X phosphate buffered saline and PBMCs were resuspended in RPMI media containing 10% fetal bovine serum.

Experiments for benzene were performed both without and with canine liver microsomes to bioactivate benzene into genotoxic metabolites (27). Pooled microsomes were prepared in our laboratory from livers obtained from healthy purpose-bred beagles (28) and checked for functional activity with a standard P450 demethylation assay (Invitrogen™ by ThermoFisher Scientific, cat.#EIAP450DMT; data not shown). Experiments were performed with 10^5^-10^6^/mL canine PBMCs in RPMI media (Fisher Scientific); each well contained 100uL of PBMCs, 5–500 uM benzene (MilliporeSigma), 60 ug of pooled canine liver microsomes, and 1 mM NADPH. Incubation times were limited to 2 h at 37 °C to minimize depletion of NADPH and liver microsomes (29).

For xylene, preliminary time course experiments with an established genotoxic human dose (200 uM) (14) showed maximal genotoxicity in canine PBMCs at 6 h (data not shown). Subsequent xylene genotoxicity experiments were performed at 6 h with 0.1–200 uM of a standard commercial mixture of xylene isomers (Fisher Scientific), along with 100 uL of a 10^5^–10^6^ /mL PBMC suspension in RMPI at 37 °C. The genotoxicity of 1,3-butadiene was not assessed *in vitro* due to safety concerns for handling 1,3-butadiene in its liquid or vapor forms (30). All *in vitro* genotoxicity experiments were performed as 4–6 replicates on 2 separate experimental days.

After dosing, PBMCs in CometChip wells was subjected to standard electrophoresis, alkaline lysis, and DNA staining with SYBR™ Gold (31). DNA was imaged with confocal microscopy (40x magnification). Comet tails, which denote DNA strand breaks, were quantified as % DNA in the comet tail using calibrated Comet Assay Analysis software (CAS; Biotechne cat. # 4260-000-CS) (32).

### Statistical analyses

Data were assessed for normal distributions using data visualization in scatter plots along with Shapiro–Wilk normality tests (appropriate for smaller sample sizes) (33). Urinary chemical concentrations between matched cases and controls at each time point were compared using Mann Whitney U tests for non-parametric data (34). Urinary chemicals between T-1y and T0 time points, across all dogs, were compared using Wilcoxon matched-pairs signed-rank tests for non-parametric data (34). The proportions of cases and controls with positive urinary cotinine were compared using Fisher’s exact tests, considering small group numbers in some categories (34). To assess a possible association between environmental tobacco smoke and urinary VOC metabolite concentrations, aggregate molar urinary VOC metabolites were also compared between urinary cotinine-positive and cotinine-negative dogs, and between dogs with and without reported specific household exposures from owner questionnaires, using Mann Whitney U tests.

To evaluate *in vitro* genotoxicity, DNA damage at each VOC concentration was compared to vehicle using one-way ANOVA for parametric data, with Dunnett’s multiple comparisons tests (34, 35). Incubations containing liver microsomes were compared to those without liver microsomes using a two-way ANOVA with Tukey’s *post hoc* test (35). The proportions of cases and controls with estimated benzene or xylene blood concentrations that reached genotoxic concentrations at either timepoint were evaluated using Fisher’s exact tests (34). All analyses were performed with commercially available software (Prism 9, GraphPad Software, San Diego CA), with *p* < 0.05 considered significant.

## Results

All five VOC metabolites were detectable in the urine of all 60 pet dogs.

### Urinary benzene metabolites

**Figure 1:**
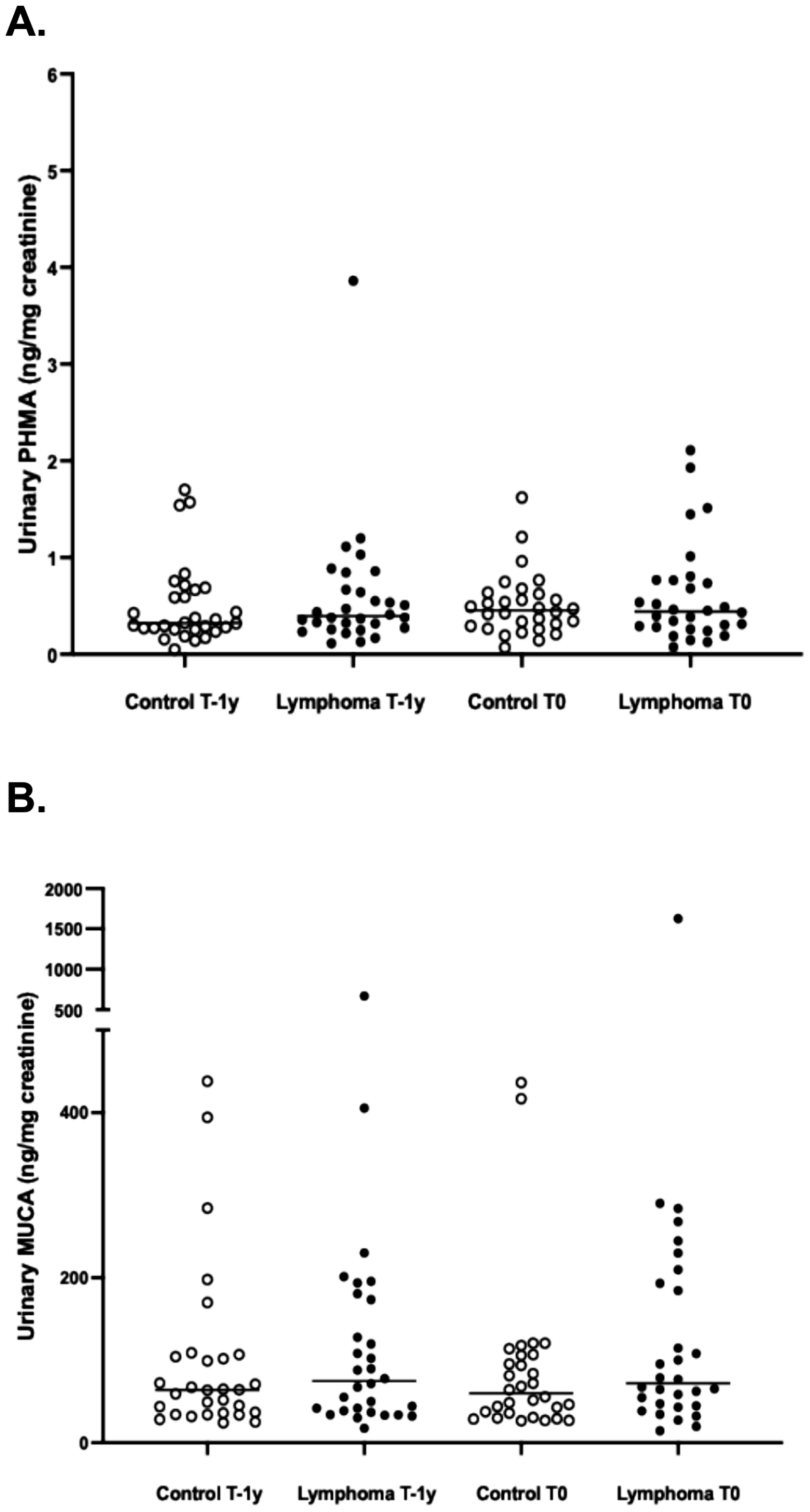
Concentrations of stable urinary benzene metabolites in golden retrievers with canine multicentric lymphoma and matched controls, measured at 2 time points: one year prior to diagnosis (T-1y) and at the time of diagnosis (or comparable time points for controls), T0. **(A)** Benzene metabolite PHMA (*N*-acetyl-*S*-(phenyl)-L-cysteine); *p* = 0.41 between cases and controls at T-1y, and *p* = 0.91 at T0. **(B)** Benzene metabolite MUCA (muconic acid); *p* = 0.45 at T-1y, and *p* = 0.22 at T0.

In the year prior to diagnosis (T-1y), urinary concentrations of the benzene metabolite PHMA were not significantly higher in cases (median 0.4 ng/mg creat) compared to matched controls (median 0.3 ng/mg creat, *p* = 0.41; Figure 1A). There were also no differences at the time of diagnosis (T0) in urinary PHMA concentrations between cases (median 0.4 ng/mg creat) and controls (median 0.5 ng/mg creat, *p* = 0.91; Figure 1A).

Consistent with these findings, there were no urinary differences in the benzene metabolite MUCA at T-1y (median 74.9 ng/mg creat for cases; 64.1 ng/mg creat for controls; *p* = 0.45), or at T0 (median 72.3 ng/mg creat for cases; median 60.1 ng/mg creat for controls; *p* = 0.22; Figure 1B). Full summary data for urinary benzene metabolites are in Supplementary Table S1.

Urinary benzene metabolite concentrations appeared stable over time across all dogs, with no detectable differences between time points for PHMA (median 0.4 ng/mg creat at T-1y, and 0.4 ng/mg creat at T0, *p* = 0.10) or for MUCA (median 66.2 ng/mg creat at T-1y and 66.6 ng/mg creat at T0, *p* = 0.63, Supplementary Table S1 and Supplementary Figure S1).

### Urinary xylene metabolites

**Figure 2:**
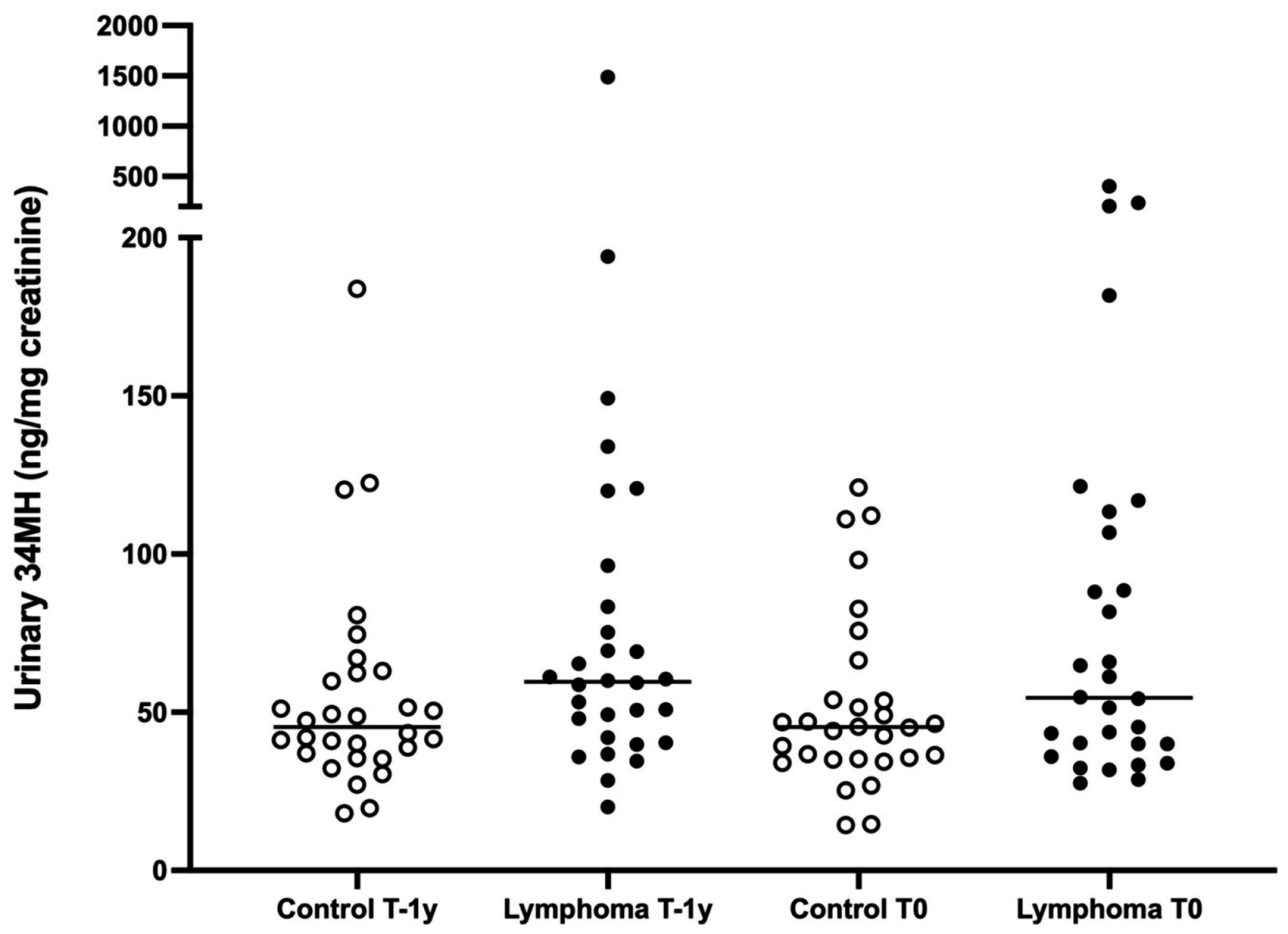
Urinary concentrations of the xylene metabolite 34MH (3-methylhippuric acid + 4-methylhippuric acid) in golden retrievers with multicentric lymphoma and matched controls, measured at two time points: 1 year prior to diagnosis (T-1y) and at the time of diagnosis (T0; or comparable time points for controls). *p* = 0.05 for T-1y and *p* = 0.12 for T0.

For xylene, urinary concentrations of its metabolite 34MH were not significantly higher in cases when compared to controls at T-1y (median 59.7 ng/mg creat for cases; 45.4 ng/mg creat for controls; *p* = 0.05) or at T0 (median 54.5 ng/mg creat for cases; 45.3 ng/mg creat for controls, *p* = 0.12; Figure 2, Supplementary Table S1). In addition, concentrations of 34MH remained stable across all dogs from T-1y (median 50.7 ng/mg creat) compared to T0 (median 46.6 ng/mg creat, *p* = 0.80, Supplementary Figure S2). We were unable to quantitate an additional xylene metabolite, *2-methylhippuric acid* (2MH) (36), because of a competing peak in canine urine.

### Urinary 1,3-butadiene metabolites

**Figure 3:**
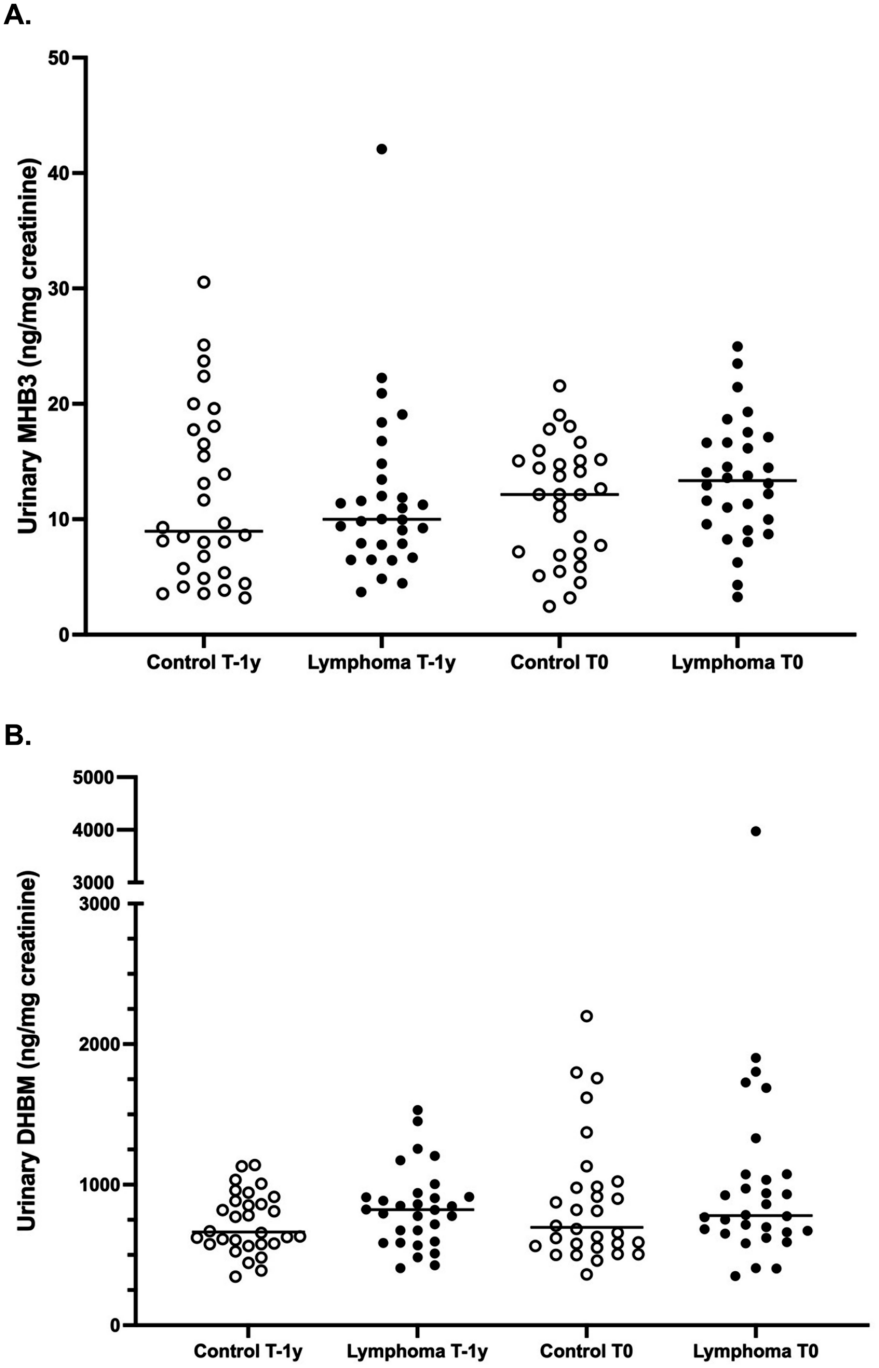
Concentrations of urinary 1,3-butadiene metabolites in golden retrievers with canine multicentric lymphoma and matched controls, measured at 2 time points: 1 year prior to diagnosis (T-1y) and at the time of diagnosis (T0; or comparable time points for controls). **(A)**: 1,3-Butadiene metabolite MHB3 (*N*-acetyl-S-(4-hydroxy-2-buten-1-yl)-L-cysteine); *p* = 0.74 for T-1y and *p* = 0.25 for T0 between cases and controls. **(B)**: 1,3-Butadiene metabolite DHBM (*N*-acetyl-S-(3,4-dihydroxybutyl)-L-cysteine); *p* = 0.19 for T-1y and *p* = 0.27 for T0 between cases and controls.

Urinary concentrations of the 1,3-butadiene metabolite MHB3 were not significantly different between groups at T-1y (median 10.0 ng/mg creat for cases; 9.0 ng/mg creat for controls; *p* = 0.74) or at T0 (median 13.4 ng/mg creat for cases; 12.2 ng/mg creat for controls; *p* = 0.25; Figure 3A). This was also true for the 1,3-butadiene metabolite DHBM at T-1y (median 822.1 ng/mg for cases; 663.7 ng/mg creat for controls; *p* = 0.19) and at T0 (median 780.3 ng/mg creat for cases; 696.5 ng/mg creat for controls; *p* = 0.27; Figure 3B).

Like benzene and xylene, urinary concentrations of 1,3-butadiene metabolites remained stable over time across all dogs (Supplementary Figure S3). Median MHB3 concentrations were 9.7 ng/mg creat at T-1y and 12.8 ng/mg creat at T0; *p* = 0.23. Similarly, median DHMB concentrations were 779.5 ng/mg creat at T-1y and 772.4 ng/mg creat at T0 (*p* = 0.11).

### Urinary cotinine

Urinary cotinine was detected overall in 6 of 30 (20%) dogs with lymphoma at one or both time points, but in no control dogs at either time point (*p* = 0.028). There were no significant differences at T-1y in aggregate molar VOC metabolites between dogs with positive cotinine (median 4.05 nmol/mg creat, range 2.83–8.14 nmol/mg creat) versus negative cotinine (median 4.02 nmol/mg creat, range 0.62–13.4 nmol/mg creat; *p* = 0.79). Similarly, at T0 there were no differences in urinary VOCs between tobacco exposure groups (cotinine-positive dogs, median 3.7 nmol/mg creat, range 2.98–5.2 nmol/mg creat; cotinine-negative dogs, median 4.3 nmol/mg creat, range 1.8–19.0 nmol/mg creat, *p* = 0.54).

### Household environmental questions

Of all 60 households in the study, natural gas or propane stoves were used for cooking in 21 dog homes (35%) while electric stoves were used in 32 (53%); 7 of 60 owners (12%) did not respond. Contrary to our hypothesis, dogs in households with electric stoves had significantly higher aggregate urinary VOC metabolite concentrations, averaged over both time points (median 4.7 nmol/mg creat, range 2.1–7.5 nmol/mg creat), compared to dogs in homes with natural gas or propane stoves (median 3.9 nmol/mg creat, range 1.9–13.5 nmol/mg creat; *p* = 0.04). Fifteen of 60 owners (25%) reported a wood burning stove or fireplace, while 39 (65%) reported neither and 6 (10%) did not answer. Aggregate urinary VOC metabolite concentrations, averaged over both time points, were not significantly higher for dogs in homes with wood burning stoves or fireplaces (median 4.3 nmol/mg creat, range 2.8–7.1 nmol/mg creat) compared to dogs in homes without (median 4.4 nmol/mg creat, range 1.9–13.5 nmol/mg creat; *p* = 0.73).

### *In vitro* genotoxic concentrations

**Figure 4:**
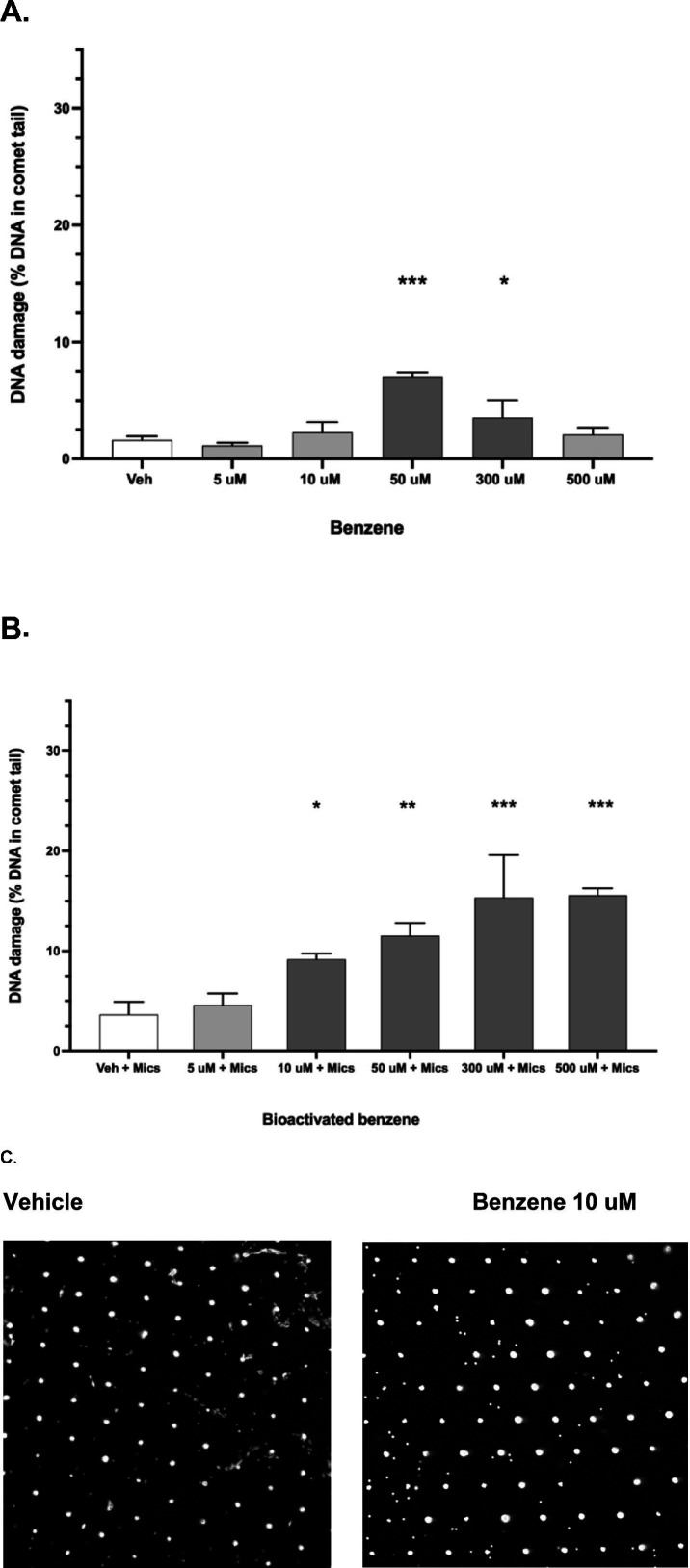
Genotoxicity of benzene (measured as % comet tail DNA representing single and double strand DNA breaks) incubated with fresh canine peripheral blood mononuclear cells (PBMC’s) for 2 h. **(A)** Benzene without added microsomes. *p ≤ 0.04; ***p < 0.0001 compared to vehicle. **(B)** Benzene with added canine liver microsomes to represent systemic bioactivation. *p < 0.03; **p < 0.002; ***p < 0.0001 compared to vehicle. DNA damage from bioactivated benzene was greater than that from benzene alone (*p* < 0.0001). Error bars are standard deviations. **(C)** Typical CometChip gel for pooled canine PBMCs exposed for 2 h to vehicle or 10 M benzene with canine liver microsomes.

**Figure 5:**
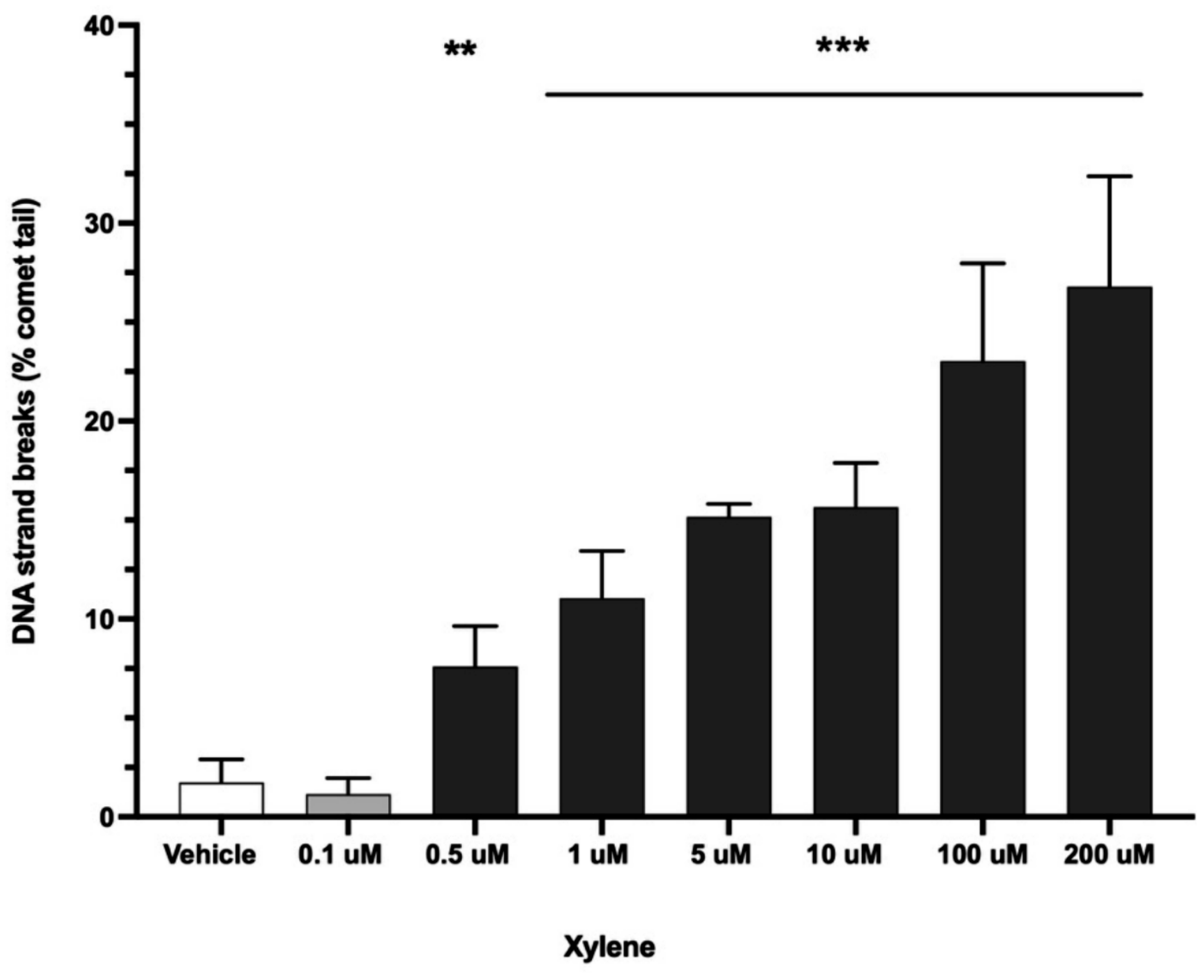
Genotoxicity of xylene (measured as % comet tail DNA representing single and double strand DNA breaks) in fresh canine peripheral blood mononuclear cells (PBMC’s). Cells were incubated with xylene for 6 h. Error bars are standard deviations. **p = 0.0003; ***p < 0.0001 compared to vehicle.

Benzene alone caused DNA strand breaks in canine PBMCs at 50 and 300 uM (Figure 4A). However, bioactivated benzene (incubated with canine liver microsomes) was more genotoxic to canine PBMCs (*p* < 0.0001), with DNA strand breaks observed at ≥ 10 uM (Figure 4B). A representative CometChip gel for vehicle control and 10 uM benzene with canine liver microsomes is shown in Figure 4C. Xylene without bioactivation showed an even lower concentration for DNA strand breaks at ≥ 0.5 uM (Figure 5).

### Estimated *in vivo* blood VOC concentrations

Estimated blood benzene concentrations across both time points, calculated using both PHMA and MUCA, ranged from 18–1,417 uM in lymphoma cases and 14–636 uM in controls. However, MUCA can arise from other sources besides benzene (37), so we amended estimated blood benzene concentrations based on PHMA alone. These amended blood benzene concentrations ranged from 1–99 uM in cases and 1–55 uM in controls over both time points (Supplementary Table S2). Based on PHMA alone, blood benzene concentrations were estimated to reach genotoxic concentrations of 10 uM in 20 of 30 dogs with lymphoma (67%) and in 16 of 30 matched controls dogs (53%; *p* = 0.43).

Estimated blood concentrations of xylene across both time points ranged from 0.1–26.4 uM in lymphoma cases and 0.2–3.7 uM in controls (Supplementary Table S2). Given the chosen reverse dosimetry assumptions, blood xylene concentrations were estimated to reach genotoxic concentrations of 0.5 uM in 26 of 30 dogs with lymphoma (87%) and in 28 of 30 matched controls dogs (93%; *p* = 0.67). Estimated blood 1,3-butadiene concentrations across both time points ranged from 3.8–27.3 uM in cases (median 14.0 uM) and 3.0–26.6 uM in controls (median 12.5 uM) (Supplementary Table S2).

## Discussion

We found evidence of likely “non-occupational” exposures to the VOCs benzene, xylene, and 1,3-butadiene in all golden retrievers that we sampled; none of these dogs were military or service dogs. Our observed concentrations for the urinary benzene metabolite PHMA ranged from 0.05–3.86 ng/mg creat (group medians 0.3–0.5 ng/mg creat), and were observably lower than urinary PHMA concentrations in non-smoking people without occupational benzene exposures (0.3–9.6 ng/mg creat; median 1.9 ng/mg creat) (38). Our observed concentrations for the urinary benzene metabolite MUCA ranged from 14.7–1,627 ng/mg creat. (medians across groups, 60–75 ng/mg creat), which is comparable to the wide range of urinary MUCA concentrations previously observed in 47 healthy dogs of various breeds (12.5–3,600 ng/mg creat, geometric mean, 96 ng/mg creat) (39). It appears that pet dogs have higher upper ranges of urinary MUCA concentrations compared to non-smoking people without occupational benzene exposures (3–461 ng/mg/creat; median 79 ng/mg/creat) (38). This could be due to higher absolute benzene exposures based on individual chewing or licking behaviors or higher relative benzene exposures based on smaller body size, but these should be reflected in the upper ranges of urinary PHMA as well. It is possible that conversion of benzene to MUCA or the urinary excretion of MUCA is more efficient in dogs than people. It is also possible that some of the observed urinary MUCA in dogs could be derived from sorbate rather than benzene (37). Potassium sorbate is an approved food preservative and is found in some dog foods (40). Because of this, urinary PHMA might be a more accurate measure of benzene exposure than MUCA in dogs.

For xylene, urinary 34MH concentrations in our study varied 100-fold, from 14.4–1,490 ng/mg creat (medians of 45–59 ng/mg creat across groups), as compared to a narrower range observed in 47 healthy dogs of various breeds (10–205 ng/mg creat; geometric mean, 35 ng/mg creat) (39). Interestingly, median urinary 34MH concentrations in human non-smokers (168 ng/mg creat) (36) are nearly 3-fold higher than median urinary 34MH values observed in our dogs. The former human study included over 7,000 participants in the National Health and Nutrition Examination Survey (NHANES); however, data on occupational exposures were not available. Generation or excretion of xylene metabolites could be more efficient in people than in dogs. Including 2MH in our reverse dosimetry calculations might have increased estimated blood xylene concentrations in dogs; however, urinary 2MH concentrations are only about 14% of total urinary xylene metabolites in people (36). Similar data are not available for dogs.

For 1,3-butadiene, urinary MHB3 ranged from 2.4–42.1 ng/mg creat (group medians of 9–13 ng/mg creat) and urinary DHMB ranged from 346 to 3,974 ng/mg creat (group medians of 664–822 ng/mg creat). In the previous study of 47 healthy dogs, urinary concentrations of MHB3 were mostly undetected, while urinary DHMB concentrations ranged widely, as in our case–control population, from 0.3–2,490 ng/mg creat (geometric mean, 115 ng/mg creat) (39). In non-smoking people, urinary MHB3 concentrations range from undetected to 122 ng/mg creat (median 21 ng/mg creat), which is comparable to the dogs in our study, but DHBM concentrations range from undetected to 582 ng/mg creat (median 105 ng/mg creat) (41), which is observably lower than dogs. Dogs might have more efficient conversion of 1,3-butadiene to DHMB than do people.

Although we found evidence of VOC exposures in virtually all pet golden retrievers sampled, we did not observe differences in urinary VOC metabolites between lymphoma cases and matched controls. While there is compelling evidence linking occupational exposures to benzene, xylene, and 1,3-butadiene with NHL in people (7, 8), there are no reports to date evaluating urinary metabolites of these VOCs in NHL for comparison with dogs.

In a previous case–control study, urinary PHMA concentrations were significantly higher in boxer dogs with lymphoma compared to age- and sex-matched controls (42). This finding could reflect enrollment bias (dogs in more urban areas might be more likely to have access to the veterinary care needed to receive a definitive diagnosis) or could reflect breed differences in disease phenotypes and subsequent environmental risks. For example, lymphoma in boxer dogs is more than 80% T-cell in origin, while lymphoma in golden retrievers is only about 50% T-cell in origin (43). It is possible that environmental risk factors vary for differing immunophenotypes of lymphoma in dogs.

Even though VOC metabolite concentrations remained relatively stable over 1 year in dogs, it would be useful to incorporate more integrated measures of ambient VOC exposures, such as silicone passive samplers (44), in future studies of lymphoma risk in dogs. It would also be useful to assess VOC exposure risk for B-cell and T-cell lymphomas individually in a larger sample size.

Significantly more golden retrievers with multicentric lymphoma had positive urinary cotinine results than control dogs, although the prevalence of urinary cotinine in cases was only 20%. This is consistent with a survey-based study that associated canine lymphoma with environmental tobacco smoke, although an unvalidated exposure index was used and controls were not matched to cases by age, breed or sex (45). Women exposed to tobacco smoke are at higher risk for NHL, particularly follicular lymphoma (46). In our dogs, aggregate VOC metabolites were not significantly higher in the small number with positive urinary cotinine (*n* = 6) compared to the rest of the study population. However, urinary cotinine was not consistent across time points in 4 of these 6 dogs, and more integrated measures of tobacco smoke exposure would be ideal. Non-tobacco sources of VOCs in dogs include indoor air pollution from gas stoves, oil and gas heaters, household paints and solvents, glues in rugs and particleboard furniture, poor ventilation, and even some artificial air fresheners, along with outdoor automobile traffic (47, 48).

An important finding of this study is that both benzene and xylene are genotoxic to canine lymphoid cells *in vitro* at micromolar concentrations. Benzene was genotoxic to healthy canine PBMCs at ≥ 10 uM in the presence of canine liver microsomes, which were included to reflect systemic bioactivation to circulating genotoxic benzene metabolites. Our results are consistent with human studies, in which major benzene metabolites were directly genotoxic to human lymphocytes at concentrations ≥ 2.6–4.7 uM (49); benzene itself was genotoxic to human lymphocytes at 50–100 uM, but lower concentrations were not evaluated (14, 50). It is important to note that benzene metabolites can be further bioactivated by lymphoid myeloperoxidases (51, 52). Activated lymphocytes increase production of myeloperoxidases, but also, conversely, show enhanced DNA repair (49). Therefore, variability in the degree of lymphocyte activation *in vitro* might influence benzene genotoxicity across studies and species.

We found that xylene was genotoxic to canine PBMCs, at concentrations ≥ 0.5 uM. In human lymphoid cells, individual xylene isomers (*o*, *m*, and *p*) caused DNA strand breaks at 50–100 uM, but lower concentrations were not tested (14). Benzene and xylene also cause oxidative stress *in vitro,* which can lead to oxidation of DNA residues (14). Subsequent studies could incorporate the enzyme FpG in comet assays, which can detect oxidized DNA residues that develop before overt strand breaks (53).

Estimated blood benzene concentrations ranged from 1 to 99 uM overall in our 60 golden retrievers. However, these estimates were dependent on human urinary excretion factors for PHMA, and toxicokinetic data for benzene are lacking in dogs. We were unable to directly measure benzene in banked blood from the GRLS cohort because samples must be collected and handled using protocols that prevent benzene loss to the air and contamination from vacutainer stoppers (54). Compared to our canine blood benzene estimates, measured blood benzene concentrations in people are much lower, ranging from 0.4–6.4 nM (24–500 ng/L) depending on smoking status (55, 56), with concentrations only as high as 28 nM in exposed workers (57). However, blood benzene is volatile and has a short half-life, and measured blood concentrations reflect only the last few minutes of exposure prior to phlebotomy (54). In contrast, urinary benzene metabolites can be elevated for days after a point exposure (54), and might represent a more accurate measure of chronic low-level exposures.

For xylene, we estimated blood concentrations of 0.1–26.4 uM across all dogs. In contrast, even though urinary 34MH concentrations are relatively higher in people than in dogs (36), measured human blood xylene concentrations are much lower, reaching up to 0.2 uM in the general population (58) and 6.7 uM in exposed workers (59). Like benzene, xylene is volatile and can be lost during blood handling but can also leach into blood samples from vacutainer stoppers (60, 61). Our reverse dosimetry assumptions were again based on human urinary excretion factors, and we might have overestimated blood xylene concentrations in dogs, even without including urinary 2MH in the calculations.

Our study has important limitations. First, our sample size was small, although we carefully matched cases to controls and focused on a single breed to minimize heritable variability. We performed a *post hoc* sample size calculation for the urinary metabolites that approached significance in this population (34MH at T-1y, *p* = 0.05). Based on the large observed variability in our population, we estimate the need for more than 280 cases and 280 controls to find the observed difference in urinary xylene metabolites, if repeatable, to be significant with *p* < 0.05 and 80% power.

For our *in vitro* genotoxicity assessments, we only measured one DNA damage endpoint, single and double stranded DNA breaks. Incorporating other DNA damage endpoints, such as oxidized DNA residues, micronuclei, or chromosomal aberrations, could provide a more comprehensive view of VOC-induced genotoxicity. We also did not assess ongoing DNA repair in canine PBMCs during chemical exposures, which could affect observed DNA damage. Further, while xylene alone was observed to cause DNA damage to canine PBMCs at low uM concentrations, including canine liver microsomes in future experiments might shift observed concentrations leading to DNA damage.

In addition, we cannot be certain that some of the DNA damage observed was due to cytotoxicity rather than direct genotoxicity. However, in human lymphocytes, the reactive benzene metabolite 1,2,4-benzenetriol does not substantially affect cell viability at 1–50 uM (91–87% viability using trypan blue dye exclusion) (13). Viability does drop to 65% at 100 uM (13). If comparable cytotoxicity held for canine cells, then cytotoxicity should not have affected our assessment of DNA damage from 10 uM benzene in the presence of activating canine liver microsomes. Xylene in combination with other VOCs was not cytotoxic to human lymphocytes at concentrations up to 200 uM (14). If comparable cytotoxicity held for canine cells, this should also have not affected the DNA damage that we observed at 0.5 uM. However, parallel cytotoxicity assessments in canine cells would have strengthened our study design.

Importantly, our reverse dosimetry estimations of blood concentrations could be inaccurate because of the lack of species-specific data for VOC dispositions in dogs. Therefore, these values should be considered exploratory estimates in dogs. Measuring benzene and xylene directly in whole blood would have provided additional information, but blood tubes must be specially processed and handled for valid blood VOC quantitation, and standard banked blood cannot be used (60, 62).

Overall, we found that virtually all pet golden retrievers sampled had evidence of exposure to benzene, xylene, and 1,3-butadiene. However, we did not find differences in estimated exposures between dogs with lymphoma and matched controls either at the time of diagnosis or 1 year prior. Subsequent studies could incorporate more integrated measures of ambient VOC exposures, such as silicone passive samplers (44), in dogs with lymphoma, and could leverage a larger sample size to examine VOC exposure risks individually for B-cell and T-cell lymphomas.

We also found that the VOCs benzene and xylene are genotoxic to canine lymphoid cells *in vitro* at micromolar concentrations, and that some pet dogs are likely exposed to genotoxic concentrations of these VOCs. Our data support recommendations to reduce VOC exposures in pet dogs. These exposures could not be attributed to environmental tobacco smoke in this small sample size, suggesting additional sources. Non-tobacco sources of VOCs in dogs include indoor air pollution from gas stoves, oil and gas heaters, household paints and solvents, glues in rugs and particleboard furniture, poor ventilation, and even some artificial air fresheners, along with outdoor automobile traffic and ambient air pollution (47, 48). Indoor air VOC concentrations can be reduced with activated carbon indoor air filtration units rated for VOC removal (63).

## Data Availability

The raw data supporting the conclusions of this article will be made available by the authors, without undue reservation.

## Supplementary material

Supplementary Table S1 Canine urinary volatile organic compound (VOC) metabolite concentrations in 30 golden retriever dogs with canine multicentric lymphoma and 30 matched unaffected controls measured at 2 time points: one year prior to diagnosis or comparable date (T-1y) and at the time of diagnosis or comparable date (T0). LOD: assay limit of detection. All five VOC metabolites were detectable in the urine of all 60 pet dogs.

Supplementary Table S2 Estimated whole blood volatile organic compound (VOC) concentrations in 30 golden retriever dogs with multicentric lymphoma and 30 matched unaffected controls measured at 2 time points: one year prior to diagnosis or comparable date (T-1y) and at the time of diagnosis or comparable date (T0). Whole blood VOC concentrations were calculated using reverse dosimetry from measured urinary VOC metabolites and should be considered estimates only. Whole blood benzene was calculated from the urinary benzene metabolite PHMA, not including the urinary benzene metabolite MUCA, which can arise from other sources.

Supplementary Figure S1. Urinary concentrations of benzene metabolites in 60 pet golden retrievers (regardless of lymphoma diagnosis) measured at 2 time points: one year prior to diagnosis (T-1y) and at the time of diagnosis (or comparable time points for controls, T0). Panel A: Urinary PHMA (N-acetyl-S-(phenyl)-L-cysteine); P = 0.10 between time points. Panel B: MUCA (muconic acid); P = 0.63 between time points.

Supplementary Figure S2. Urinary concentrations of the xylene metabolite 34MH (3-methylhippuric acid + 4-methylhippuric acid) in 60 pet golden retrievers (30 cases of multicentric lymphoma and 30 unaffected controls) measured at 2 time points: one year prior to diagnosis (T-1y) or at the time of diagnosis (or comparable time points for controls; T0). P 869 = 0.80 between time points.

Supplementary Figure S3. Urinary concentrations of 1,3-butadiene metabolites in 60 pet golden retrievers (regardless of lymphoma diagnosis) measured at 2 time points: one year prior to diagnosis (T-1y) and at the time of diagnosis (or comparable time points for controls; T0). Panel A: MHB3 (N-acetyl-S-(4-hydroxy-2-buten-1-yl)-L-cysteine); P = 0.23 between time points. Panel B: DHBM (N-acetyl-S-(3,4-dihydroxybutyl)-L-cysteine); P = 0.11 between time points.
