# Supplementary figures and images for: Genotoxic exposures to volatile organic compounds in golden retrievers with and without multicentric lymphoma
Source: Front Vet Sci. 2026 Apr 13;13:1783854. doi: 10.3389/fvets.2026.1783854 (PMC13111026; doi:10.3389/fvets.2026.1783854)

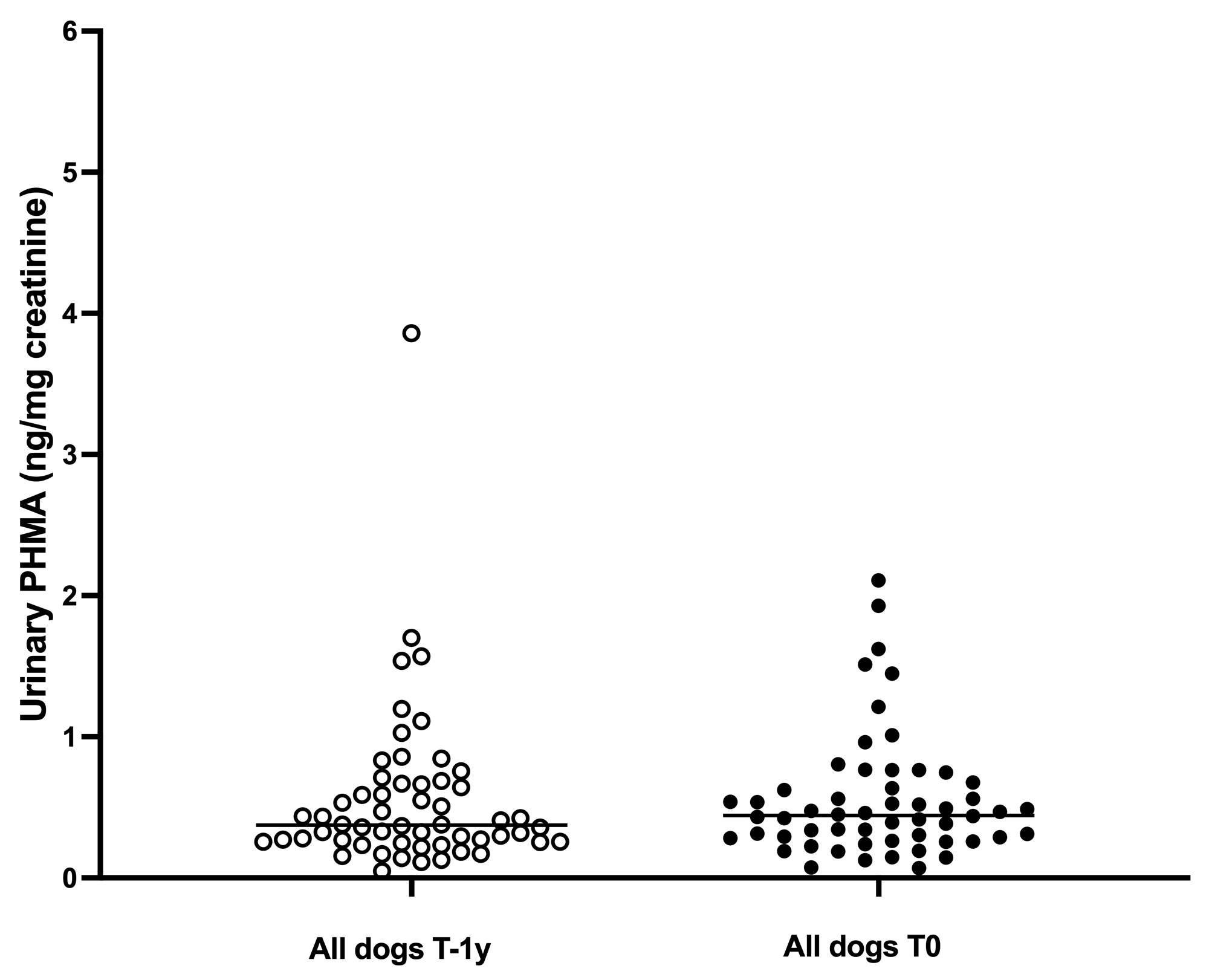

Supplement: Supplementary file 1 [file Supplementary_file_1.zip › Supplementary Material/Figure S1A.JPEG]

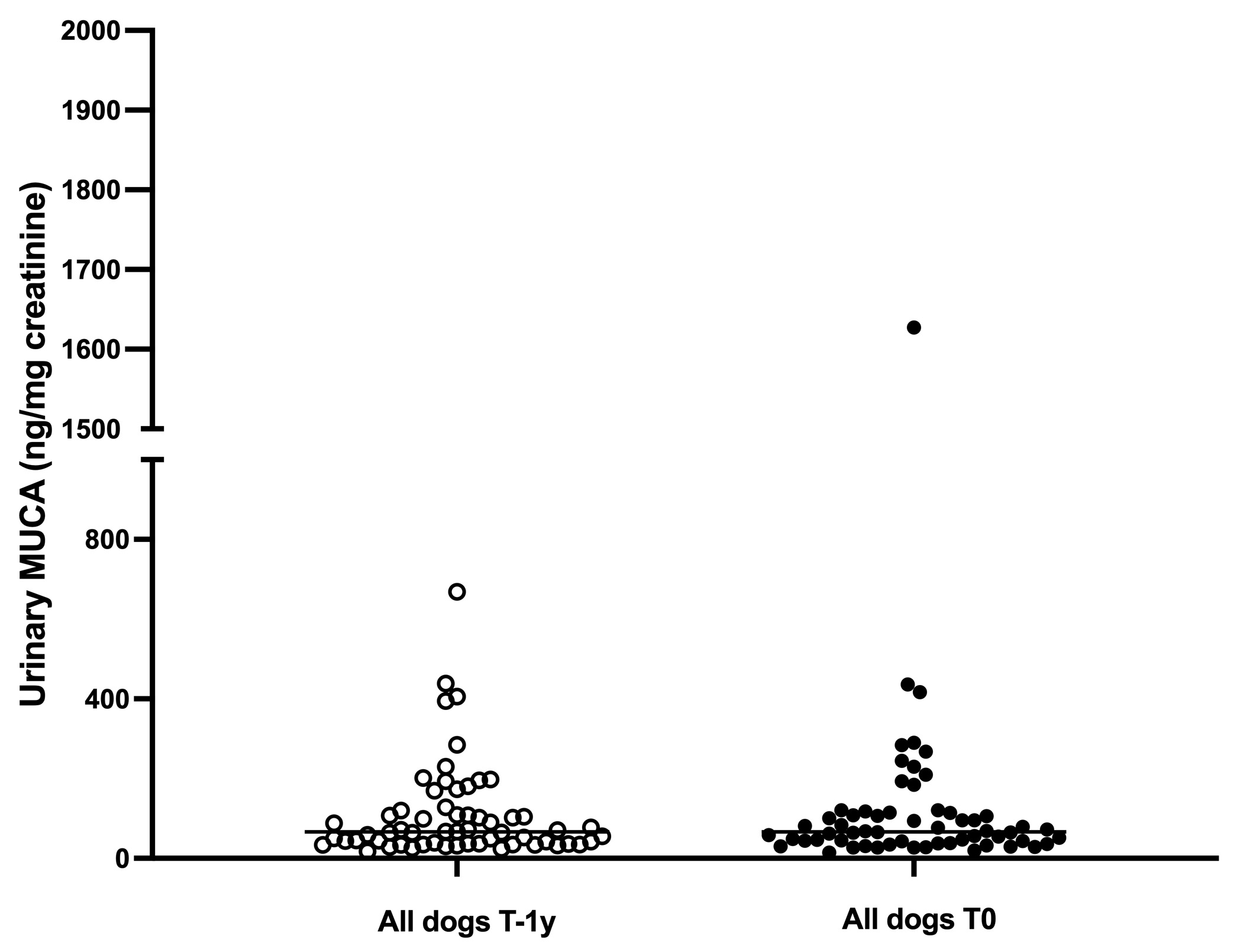

Supplement: Supplementary file 1 [file Supplementary_file_1.zip › Supplementary Material/Figure S1B.JPEG]

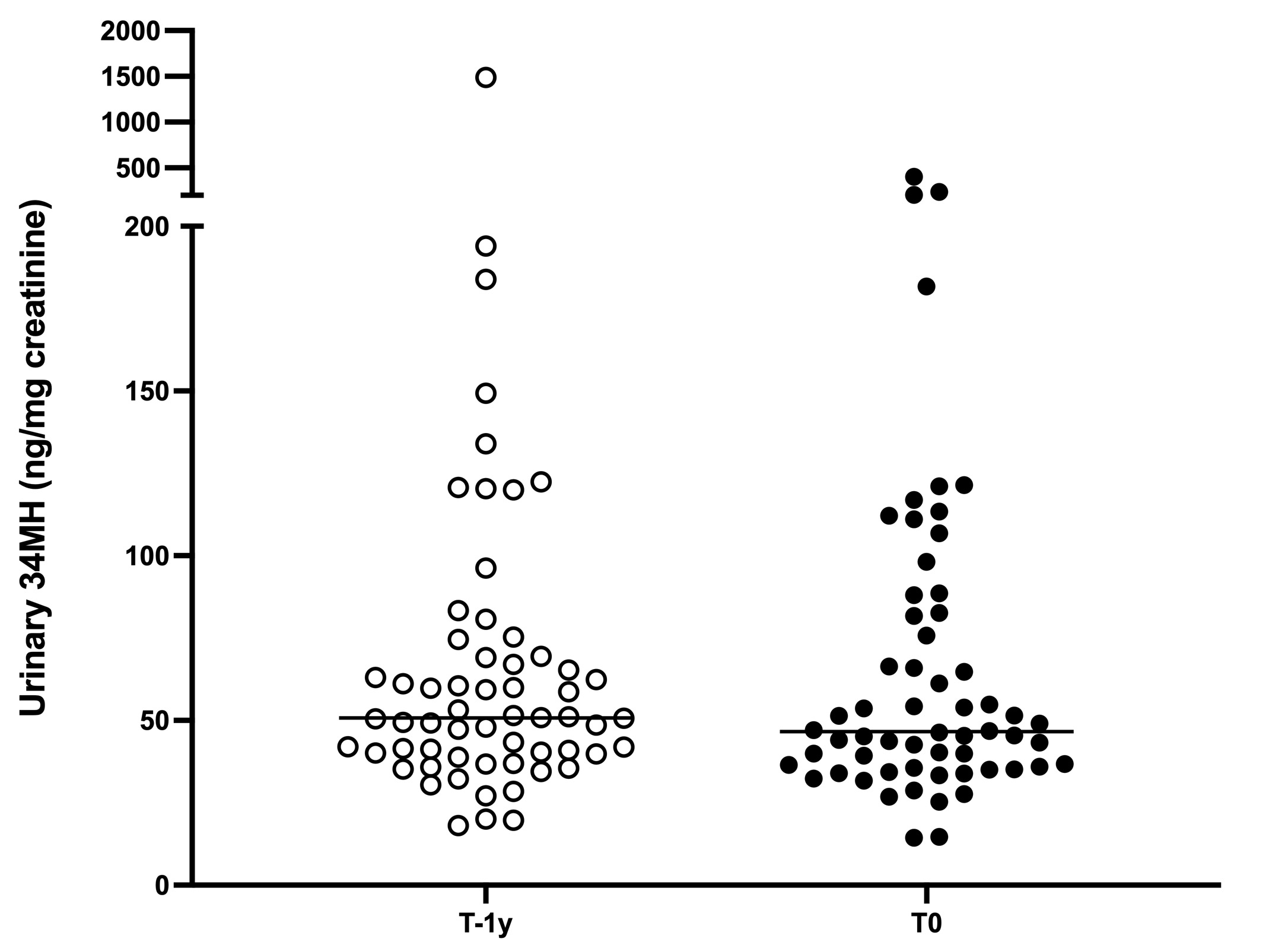

Supplement: Supplementary file 1 [file Supplementary_file_1.zip › Supplementary Material/Figure S2.JPEG]

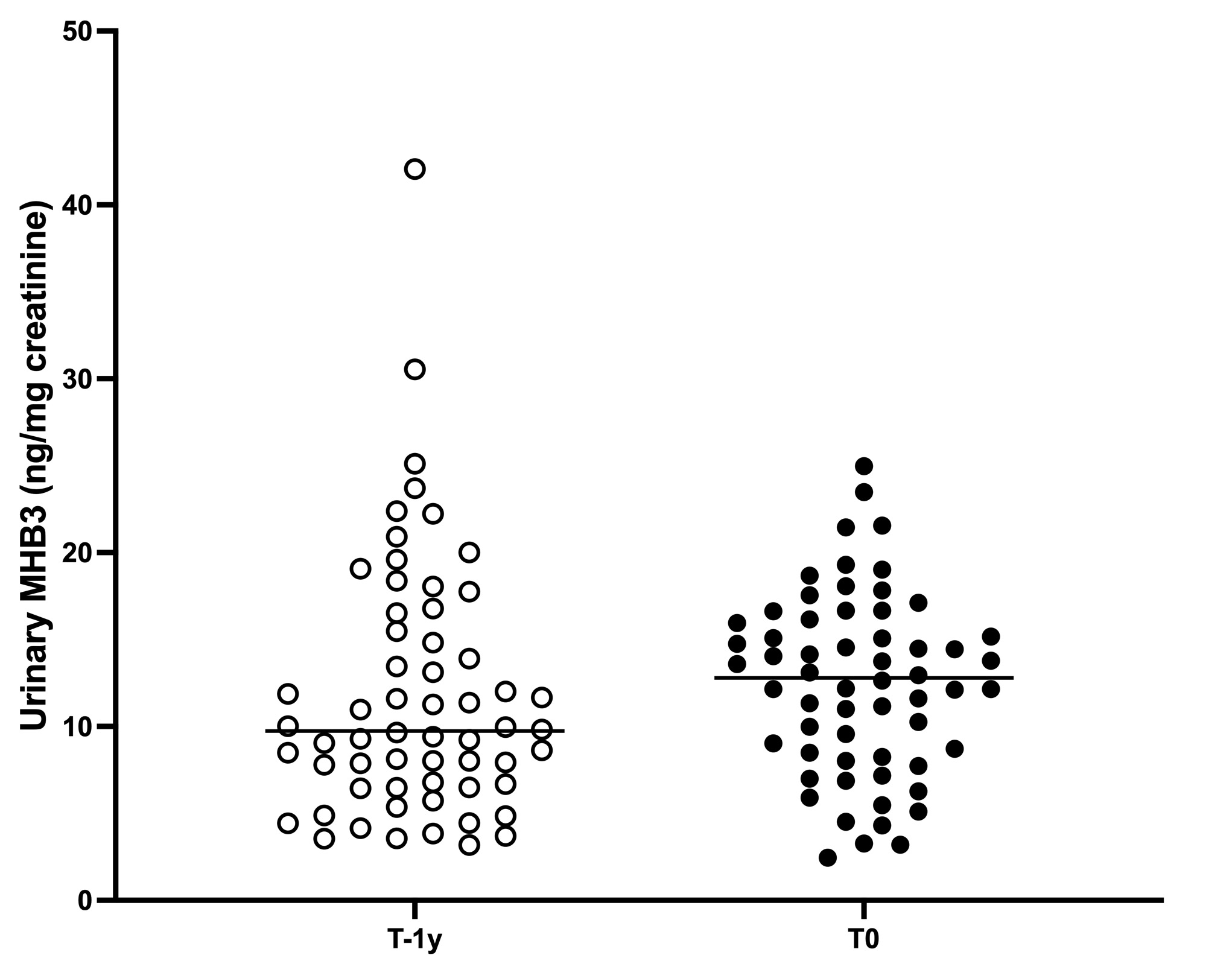

Supplement: Supplementary file 1 [file Supplementary_file_1.zip › Supplementary Material/Figure S3A.JPEG]

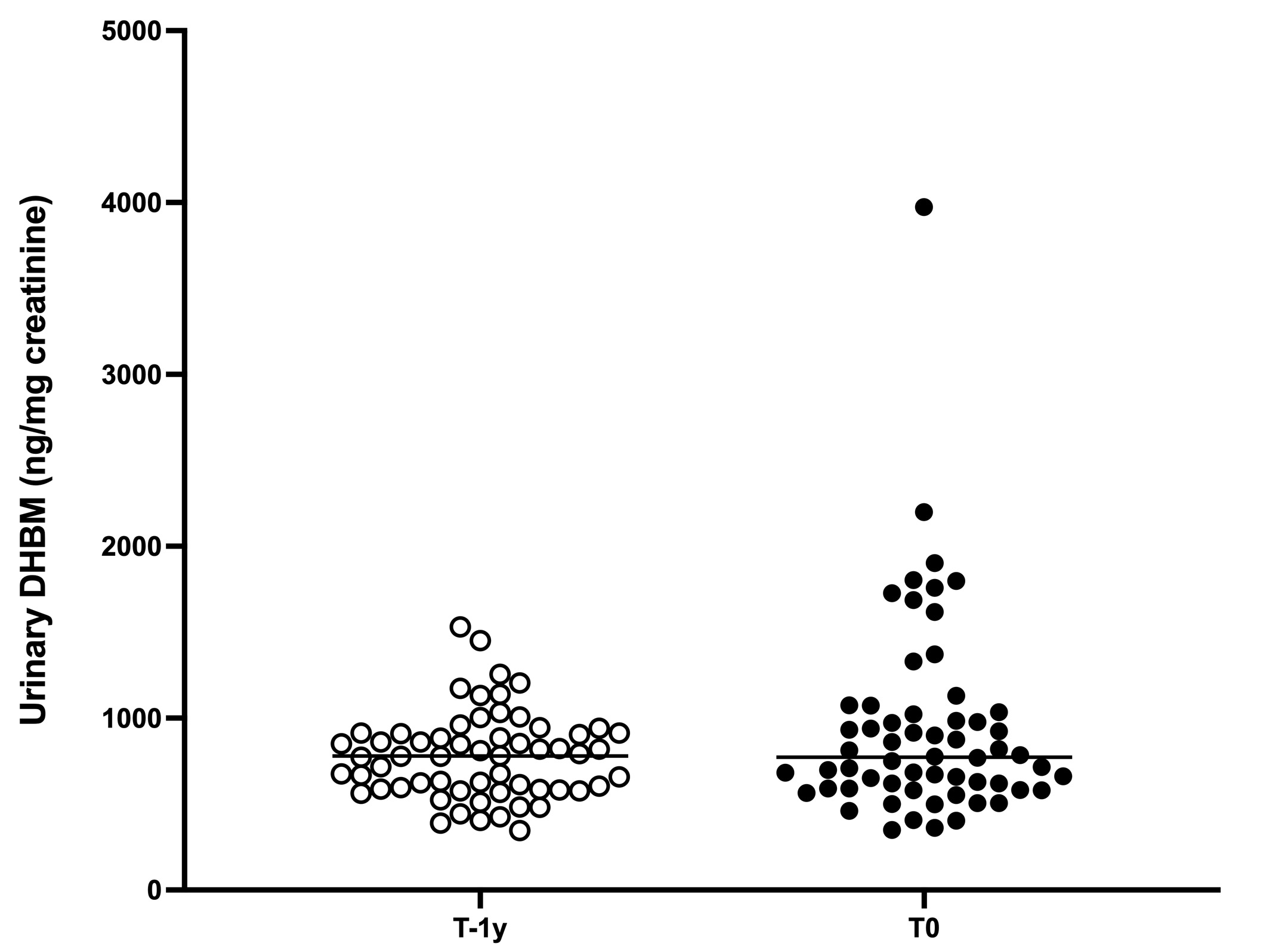

Supplement: Supplementary file 1 [file Supplementary_file_1.zip › Supplementary Material/Figure S3B.JPEG]
