# Supplementary material for: Genotoxic exposures to volatile organic compounds in golden retrievers with and without multicentric lymphoma
Source: Front Vet Sci. 2026 Apr 13;13:1783854. doi: 10.3389/fvets.2026.1783854 (PMC13111026; doi:10.3389/fvets.2026.1783854)
Supplement: Supplementary Table S1 — Canine urinary volatile organic compound (VOC) metabolite concentrations in 30 golden retriever dogs with canine multicentric lymphoma and 30 matched unaffected controls measured at 2 time points: one year prior to diagnosis or comparable date (T-1y) and at the time of diagnosis or comparable date (T0). LOD: assay limit of detection. All five VOC metabolites were detectable in the urine of all 60 pet dogs. [file Table_S1.docx]

**Supplemental Table S1:** Canine urinary volatile organic compound (VOC) metabolite concentrations in 30 golden retriever dogs with canine multicentric lymphoma and 30 matched unaffected controls measured at 2 time points: one year prior to diagnosis or comparable date (T-1y) and at the time of diagnosis or comparable date (T0). LOD: assay limit of detection. All five VOC metabolites were detectable in the urine of all 60 pet dogs.

| **Parent compound** | **Measured urinary metabolite** | **Lymphoma cases**  **Median (range)** | **Unaffected controls**  **Median (range)** | **P value between groups** |
| --- | --- | --- | --- | --- |
| Benzene | PHMA  (LOD: 0.150 ng/mL) | *T-1y Lymphoma*: 0.4 ng/mg creat  (0.1-3.9 ng/mg creat)  *T0 Lymphoma*: 0.4 ng/mg creat  (0.1-2.1 ng/mg creat) | *T-1y Control*: 0.3 ng/mg creat  (0.05-1.7 ng/mg creat)  *T0 Control*: 0.5 ng/mg creat  (0.1-1.6 ng/mg creat) | 0.42  0.78 |
|  | MUCA  (LOD: 9.81 ng/mL) | *T-1y Lymphoma*:  74.9 ng/mg creat  (17.8-668.4 ng/mg creat)  *T0 Lymphoma*:  72.3 ng/mg creat  (14.7-1627.0 ng/mg creat) | *T-1y Control*:  64.1 ng/mg creat  (24.5-438.1 ng/mg creat)  *T0 Control*:  60.1 ng/mg creat  (26.8-436.3 ng/mg creat) | 0.50  0.18 |
| Xylene | 34MHA  (LOD: 8.00 ng/mL) | *T-1y Lymphoma*:  59.7 ng/mg creat  (20.1-1490 ng/mg creat)  *T0 Lymphoma*:  54.5 ng/mg creat  (27.6-403.4 ng/mg creat) | *T-1y Control*:  45.4 ng/mg creat  (18.1-183.8 ng/mg creat)  *T0 Control*:  45.3 ng/mg creat  (14.4-121.0 ng/mg creat) | 0.09  0.07 |
| 1,3-Butadiene | MHB3  (LOD: 0.600 ng/mL) | *T-1y Lymphoma*:  10.0 ng/mg creat  (3.7-42.1 ng/mg creat)  *T0 Lymphoma*:  13.4 ng/mg creat  (3.3-25.0 ng/mg creat) | *T-1y Control*:  9.0 ng/mg creat (3.2-30.6 ng/mg creat)  *T0 Control*:  12.2 ng/mg creat  (2.4-21.6 ng/mg creat) | 0.86  0.26 |
|  | DHBM  (LOD: 5.25 ng/mL) | *T-1y Lymphoma*: 822.1 ng/mg creat  (405.7-1531.0 ng/mg creat)  *T0 Lymphoma*: 780.3 ng/mg creat  (349.7-3974.0 ng/mg creat) | *T-1y Control*: 663.7 ng/mg creat  (345.8-1139.0 ng/mg creat)  *T0 Control*: 696.5  ng/mg creat  (361.2-2199.0 ng/mg creat) | 0.15  0.50 |
