# Supplementary material for: Genotoxic exposures to volatile organic compounds in golden retrievers with and without multicentric lymphoma
Source: Front Vet Sci. 2026 Apr 13;13:1783854. doi: 10.3389/fvets.2026.1783854 (PMC13111026; doi:10.3389/fvets.2026.1783854)
Supplement: Supplementary Table S2 — Estimated whole blood volatile organic compound (VOC) concentrations in 30 golden retriever dogs with multicentric lymphoma and 30 matched unaffected controls measured at 2 time points: one year prior to diagnosis or comparable date (T-1y) and at the time of diagnosis or comparable date (T0). Whole blood VOC concentrations were calculated using reverse dosimetry from measured urinary VOC metabolites and should be considered estimates only. Whole blood benzene was calculated from the urinary benzene metabolite PHMA, not including the urinary benzene metabolite MUCA, which can arise from other sources. [file Table_S2.docx]

**Supplemental Table S2**: Estimated whole blood volatile organic compound (VOC) concentrations in 30 golden retriever dogs with multicentric lymphoma and 30 matched unaffected controls measured at 2 time points: one year prior to diagnosis or comparable date (T-1y) and at the time of diagnosis or comparable date (T0). Whole blood VOC concentrations were calculated using reverse dosimetry from measured urinary VOC metabolites and should be considered estimates only. Whole blood benzene was calculated from the urinary benzene metabolite PHMA, not including the urinary benzene metabolite MUCA, which can arise from other sources.

| **Parent compound** | **Lymphoma cases**  **Median (range)** | **Unaffected controls**  **Median (range)** |
| --- | --- | --- |
| Benzene | *T-1y Lymphoma*: 10.8 uM  (3.3-98.5 uM)  *T0 Lymphoma*:  9.1 uM  (0.8-79.8 uM) | *T-1y Control*: 7.4 uM  (1.5-42.3 uM)  *T0 Control*: 8.6 uM  (1.5-54.8 uM) |
| Xylene | *T-1y Lymphoma*:  1.1 uM  (0.4-26.4 uM)  *T0 Lymphoma*:  0.8 uM  (0.1-11.6 uM) | *T-1y Control*:  0.8 uM  (0.2-3.4 uM)  *T0 Control*:  0.7 uM  (0.2-3.7 uM) |
| 1,3-Butadiene | *T-1y Lymphoma*:  5.3 uM  (2.0-15.4 uM)  *T0 Lymphoma*:  8.3 uM  (1.2-15.7 uM) | *T-1y Control*:  4.4 uM  (1.1-10.3 uM)  *T0 Control*:  7.3 uM  (1.6-16.7 uM) |
|  | *T-1y Lymphoma*: 822.1 ng/mg creat  (405.7-1531.0 ng/mg creat)  *T0 Lymphoma*: 780.3 ng/mg creat  (349.7-3974.0 ng/mg creat) | *T-1y Control*: 663.7 ng/mg creat (345.8-1139.0 ng/mg creat)  P = 0.15  *T0 Control*: 696.5 ng/mg creat  (361.2-2199.0 ng/mg creat)  P = 0.50 |
